# Transcriptional Regulation of Frizzled-1 in Human Osteoblasts by Sp1

**DOI:** 10.1371/journal.pone.0163277

**Published:** 2016-10-03

**Authors:** Shibing Yu, Laura M. Yerges-Armstrong, Yanxia Chu, Joseph M. Zmuda, Yingze Zhang

**Affiliations:** 1 Department of Medicine, School of Medicine, University of Pittsburgh, Pennsylvania, United States of America; 2 Department of Epidemiology, Graduate School of Public Health, University of Pittsburgh, Pennsylvania, United States of America; 3 Department of Human Genetics, Graduate School of Public Health, University of Pittsburgh, Pennsylvania, United States of America; 4 Program in Personalized and Genomic Medicine and Department of Medicine, Division of Endocrinology, Diabetes and Nutrition, School of Medicine, University of Maryland, College Park, Maryland, United States of America; Universitat des Saarlandes, GERMANY

## Abstract

The wingless pathway has a powerful influence on bone metabolism and is a therapeutic target in skeletal disorders. Wingless signaling is mediated in part through the Frizzled (FZD) receptor family. FZD transcriptional regulation is poorly understood. Herein we tested the hypothesis that Sp1 plays an important role in the transcriptional regulation of FZD1 expression in osteoblasts and osteoblast mineralization. To test this hypothesis, we conducted *FZD1* promoter assays in Saos2 cells with and without Sp1 overexpression. We found that Sp1 significantly up-regulates *FZD1* promoter activity in Saos2 cells. Chromatin immunoprecipitation (ChIP) and electrophoretic mobility shift (EMSA) assays identified a novel and functional Sp1 binding site at -44 to -40 from the translation start site in the FZD1 promoter. The Sp1-dependent activation of the *FZD1* promoter was abolished by mithramycin A (MMA), an antibiotic affecting both Sp1 binding and Sp1 protein levels in Saos2 cells. Similarly, down-regulation of Sp1 in hFOB cells resulted in less FZD1 expression and lower alkaline phosphatase activity. Moreover, over-expression of Sp1 increased FZD1 expression and Saos2 cell mineralization while MMA decreased Sp1 and FZD1 expression and Saos2 cell mineralization. Knockdown of FZD1 prior to Sp1 overexpression partially abolished Sp1 stimulation of osteoblast differentiation markers. Taken together, our results suggest that Sp1 plays a role in human osteoblast differentiation and mineralization, which is at least partially mediated by Sp1-dependent transactivation of FZD1.

## Introduction

Transcription factor Sp1 regulates genes in both a positive and negative manner [1]. Sp1 plays an important role in cell cycle progression [2,3], apoptosis [4,5], and the cellular response to hormone/growth factor stimulation [6,7]. Sp7 (osterix), another member of the Sp transcription factor family, is essential for bone development and mineralization [8]. Knockout of Sp7 leads to a significant delay and reduction of bone maturation and mineralization in newborn mice [8]. Although a direct role of Sp1 in osteoblast differentiation and bone formation is less well known, a single nucleotide polymorphism (SNP) affecting Sp1 binding in the COL1A1 gene promoter has been associated with reduced bone mineral density (BMD) [9] and increased risk of osteoporotic fracture [10–14]. These studies support a potential role of Sp1 in osteoblast differentiation and mineralization.

Frizzled1 (FZD1) is a receptor for the Wnt signaling pathway and promoter variants in FZD1 have been associated with BMD [15,16]. FZD1 plays a role in osteoblast mineralization and the *FZD1* promoter is regulated by several transcription factors including early growth response 1 (EGR1), E2F transcription factor 1 (E2F1) and activating protein 2 (TFAP2) [15,17,18]. In addition, allele specific transactivation of the FZD1 promoter by EGR1 has also reported [15].

To further investigate the transcriptional regulation of *FZD1*, we performed bioinformatics analysis *in silico* and identified putative binding sites for Sp1 in the FZD1 promoter. To determine whether Sp1 is a regulator of osteoblast mineralization and FZD1 expression, we analyzed the transactivation of the *FZD1* promoter by Sp1 and the effects of Sp1 on osteoblast mineralization in Saos2 cells and further validated in human fetal osteoblasts (hFOB). Saos2 is a cell line derived from primary osteosarcoma and has been well documented for the natural manner of osteoblastic differentiation [19,20], therefore we used Saos2 as our *in vitro* osteoblast mineralization model. We identified a novel functional Sp1 binding site and its role in the activation of *FZD1* promoter. Furthermore, Sp1 enhanced mineralization of Saos2 osteoblastic cells at a later stage of osteoblast differentiation. Our findings suggest that Sp1 regulates *FZD1* gene expression and influences mineralization of human osteoblasts.

## Materials and Methods

### Construction of plasmid and luciferase assay

Luciferase reporter plasmids of pGL3 basic (Promega, USA) containing 726 base pair (bp, full length -655 to +71 nucleotide relative to the translation start site) or 246 bp (proximal -175 to +71 nucleotide relative to the translation start site) *FZD1* promoter fragments (FZD1-pGL3 plasmids) were described previously [15,17] and used for transfection. Recombinant plasmids containing mutated nucleotides AAA in each of the two putative core Sp1 banding site (-44 to -40 and -97 to -93 nucleotide relative to the translation start site), were generated using the wild type proximal FZD1-pGL3 plasmid and the Quikchange lightning site directed mutagenesis kit (Agilent Technologies, USA). Mutation was confirmed by direct sequencing and the plasmids were used for transfection and luciferase assay. Expression plasmids for Sp1 and mutated Sp1 were purchased from Addgene (#12097 and #12098, respectively).

For transfection experiments, Saos2 cells were seeded at the density of 1x 10^5^/well in 24-well plates for 24 hr, followed by co-transfection of 100 ng *FZD1* reporter plasmid and 250 ng expression plasmid for Sp1. Co-transfection of *FZD1* reporter and β-gal expression plasmid was used as a control. A renilla luciferase reporter was included as an internal control for all transfections. Transfected cells were cultured for another 48 hr and whole cell protein was harvested in 1x passive lysis buffer for luciferase assay. Dual luciferase activity was measured on a SpectraMax L microplate reader (Molecular Device, USA) using a dual luciferase assay kit (Promega, USA). Luciferase assay was carried out in triplicate and repeated three times.

### Chromatin immunoprecipitation (ChIP) assay

ChIP assay was performed as described [17,18]. In brief, Saos2 cells were treated with 1% formaldehyde freshly made in PBS for 10 min at room temperature. Chromatin sample was prepared and subjected to ChIP assay with antibodies against Sp1 or normal IgG as a control. Pull-down DNA fragments and input DNA were used as templates for PCR using primers designed to amplify -273 to +54 or -44 to +54 region relative to the translation start site of the *FZD1* promoter containing the putative Sp1 binding sites. PCR products were analyzed using agarose electrophoresis.

### Electrophoretic mobility shift assay (EMSA)

Sense and antisense oligonucleotides containing putative Sp1 core binding site were synthesized (CCGCCGGCCGTGCCCCTGGCAGCC, with Sp1 binding site underlined), end-labeled with biotin and annealed. Saos2 cells were infected with Adenovirus-Sp1 (Applied Biological Materials Inc) for 48 hr, and nuclear extracts were prepared using a Nuclear Extraction Kit (Active Motif, USA). Two microgram nuclear protein was used for each binding reaction with the Sp1 binding site oligonucleotides and EMSA experiments were carried out using a Lightshift Chemiluminescent EMSA kit according to the manufacturer's instructions (Thermo Scientific, USA). For Sp1 supershift experiments, 0.2 μg of Sp1 antibody (Santa Cruz, sc-59) was added to the reaction and incubated for an additional 60 min at 4°C following the standard binding reaction. Normal rabbit IgG was used in parallel as a control. EMSA assay was repeated three times.

### Cell culture and mithramycin A (MMA) treatment

Saos2 cells were seeded at the density of 4x10^5^ cells/35 mm dish and cultured for 24 hr, followed by MMA treatments for another 48 hr at 20, 100 and 200 nM (100 μM stock was prepared in 100% ethanol). The MMA treatment assay was repeated two times.

Western blot

Western blot was conducted as described [17,18] using 20μg protein from whole cell lysate. Membranes were blotted with the following primary antibodies Sp1 (Santa Cruz Biotech, USA, sc-59) and FZD1 (Abgent, USA, AP2755b).

### Gene knockdown by siRNA in osteoblast

Human fetal osteoblast cells hFOB and Saos2 cells (1 x 10^5^ cells /well) were seeded in 12 well plates for overnight prior to transfection. Sp1 siRNA (50 nM) or an equal amount of scramble siRNA was transfected into Saos2 and hFOB cells. At 48 hr post transfection, the cells were harvested for further analysis including Western blot, real-time quantitative PCR and alkaline phosphatase (ALP) staining. FZD1 knockdown experiments were performed as described [17].

### Real-time quantitative PCR

Human FOB cells were transfected with 50 nM Sp1 siRNA or scramble siRNA and cultured for an additional 48 hr. The cells were harvested for total RNA isolation using TRIzol reagent (Invitrogen, USA). One microgram of total RNA was used for reverse transcription using High Capacity cDNA Reverse Transcriptase kit (Life Technology, USA). Real-time quantitative PCR was performed using 10 ng cDNA and SYBR green master kit and carried out on QuantStudio™ 5 System (Applied Biosystems, USA). PCR primers for FZD1, ALP, COL1A, OCN and OPN genes are described [17,18]. The expression levels of Sp1 were determined using PCR primer pair 5'-CCGCAGGTGAGAGGTCTTG-3' /5'-ACAGCCCAGATGCCCAACC-3'.

Saos2 cells were transfected with 20 μM of FZD1 siRNA or scramble siRNA and cultured for 24 hr. The cells were subsequently transfected with 1μg of Sp1 or β-gal expression plasmid and cultured for an additional 48 hr. Cells were harvested for total RNA isolation and real-time quantitative PCR analysis as described above.

### Alkaline phosphatase staining in osteoblast

Saos2 and hFOB cells were seeded and transfected with siRNA as described above. At 48 hr post transfection, growth media were changed and cells were cultured for an additional 72 hr. Cells were then fixed with 4% formaldehyde at room temperature for 10 min followed by an incubation with BCIP/NBT liquid substrate solution (Sigma, USA) at room temperature for 15 min. Stained cells were photographed and ALP activity (staining intensity) were determined by densitometry using ImageJ software (https://imagej.nih.gov/ij/index.html). ALP activity experiments were conducted in triplicates and repeated at least 2 times independently.

### Mineralization and Alizarin red S staining

Saos2 cells were cultured in osteoblastic differentiation medium containing 50 μg/ml ascorbic acid and 10 mM 3-glycerophosphate for up to 18 days. Differentiation medium was changed every other day. For the Sp1 over-expression experiment, cells were infected with Adenovirus-Sp1 or Adenovirus-β-gal for 48 hr and then cultured in differentiation medium for 9 and 18 days to determine the effects of Sp1 in early and late stage of differentiation and mineralization. For the MMA treatment, 100 nM MMA or equal volume of ethanol was added to the MMA treatment or control group, respectively. After 24 hr, the cells were cultured in the same differentiation condition as described above and fixed at day 14 for mineralization assay. Cells were also cultured in growth media without the supplements in parallel as an undifferentiated control for both experiments. Cell fixing and staining with Alizarin red-S and quantification of mineralization in these cells using cetylpyridinium chloride was performed as described [18].

### Statistical analysis

Statistical analysis was performed using Student's t-test or one-way ANOVA followed by a Bonferroni multiple comparison adjustment. Differences were considered significant at *P <0*.*05*.

## Results

### Sp1 up-regulates *FZD1* promoter activity

**Fig 1:**
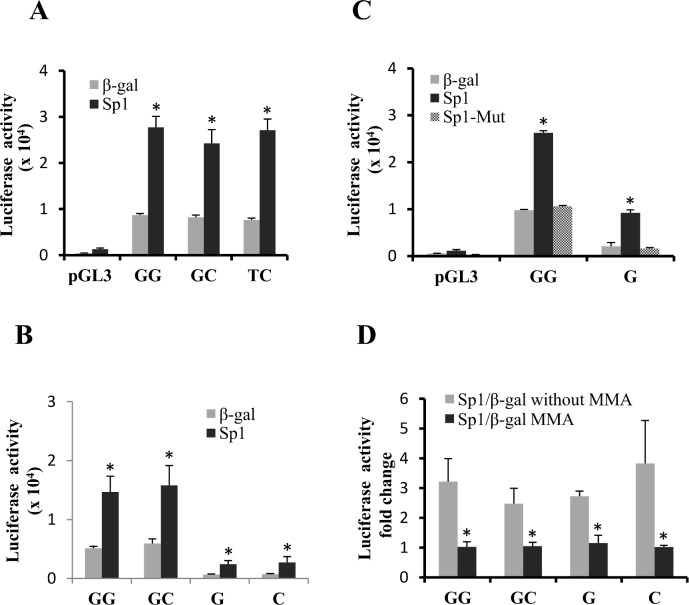
Sp1 up-regulates *FZD1* promoter activity in Saos2 cells. (A) Increase in haplotype-specific *FZD1* promoter activity by Sp1 overexpression. Saos2 cells were transfected with 0.1 μg GG (wild type), GC or TC haplotype specific *FZD1* promoters in the presence or absence of 0.25μg pCMV/Sp1 expression plasmid. After 36 hr, cells were harvested and used for dual luciferase assays. The p-values for direct comparisons between β-gal and AP2 treated promoters were calculated for each haplotype specific plasmid. (B) Increase in *FZD1* promoter by Sp1 is retained in the proximal promoter. Saos2 cells were transfected with 0.1μg of full length promoters (containing both rs2232157 and rs2232158) or proximal rs2232158-G or -C allele promoters with or without 0.25 μg of pCMV/Sp1 expression plasmid. After 36 hr, cells were harvested for the dual luciferase assays. (C) The effects of mutant Sp1 proteins on *FZD1* promoter activity. Full length wild type promoter (-GG) or proximal rs2232158-G allele promoter were co-transfected with or without 0.25 μg of wild Sp1 and mutant SP1 expression plasmids in Saos2 cells. Dual luciferase assay was processed. The p-values for direct comparisons between β-gal and Sp1 treated promoters were calculated for each mutant plasmid. (D) Suppression of Sp1 activated *FZD1* promoter activity by MMA. Full length promoter or proximal rs2232158-G and–C allele-specific promoters were co-transfected with or without 0.25 μg of Sp1 expression plasmids in Saos2 cells for 8 hours, followed by MMA or vehicle (100% ethanol) treatment for additional 36 hours. Dual luciferase assay was processed and p-values for direct comparisons between β-gal and Sp1 treated promoters were calculated for each plasmid. For all experiments, 10 ng pRL-null reporter was used as an internal control. * indicates significant differences in promoter activity between Sp1 and β-gal (*P* < 0.05).

Our previous studies reported that several transcription factors (EGR1, E2F1 and TFAP2) regulate FZD1 promoter activity in osteoblasts and that regulation by Egr1 was modulated by a promoter polymorphism (rs2232158). Moreover, both rs2232157 and rs2132158 in the FZD1 promoter have been associated with bone mineral density (BMD) [15,16]. Bioinformatics analysis of rs2232157 identified a putative Sp1 binding site on the antisense sequences for each of the alleles (C/AGGGCGCGC) (PROMO transcription factor site search engine (http://alggen.lsi.upc.es/cgi-bin/promo_v3/promo/promoinit.cgi?dirDB=TF_8.3) and the sequences associated with the T allele had a better match to the binding site matrix compared to the wild type G allele. To test whether Sp1 regulates FZD1 promoter activity and whether this effect is modified by rs3322157, we co-transfected Sp1 expression plasmids with rs2232157 and rs2232158 haplotype specific pGL3-FZD1 reporters and analyzed the promoter activity. Overexpression of Sp1 significantly increased FZD1 promoter activity approximately 3-fold for all three naturally occurring haplotypes (Fig 1A). However, there were no significant differences in Sp1-dependent promoter transactivation for the GC and TC haplotypes corresponding to the G and T allele of rs2232157, respectively. Furthermore, all three haplotypes had similar increases in promoter activity in cells overexpressing Sp1, suggesting that the Sp1 transactivation is independent of the two promoter SNPs.

To determine the specific regions responsible for the Sp1 transactivation of FZD1, we tested Sp1 effects on promoter activity of both full length (726 bp) and proximal (246 bp) FZD1 promoter reporters. Overexpression of Sp1 produced similar increases (approximately 4-fold) for both promoters regardless of the promoter length and G or C alleles of rs2232158 (Fig 1B). Transactivation was abolished for the full length and proximal promoters when a loss of function Sp1 mutant was overexpressed (Fig 1C), suggesting that the proximal promoter is responsible for Sp1 activation. Furthermore, treating the Sp1 overexpressing cells with a known inhibitor of Sp1 activity and Sp1 protein levels, Mithramycin A (MMA), partially abolished the Sp1 activation of the FZD1 promoters (Fig 1D). These results suggest that Sp1 transactivates the FZD1 promoter through the proximal region of the promoter.

### Direct binding of Sp1 to the *FZD1* promoter

**Fig 2:**
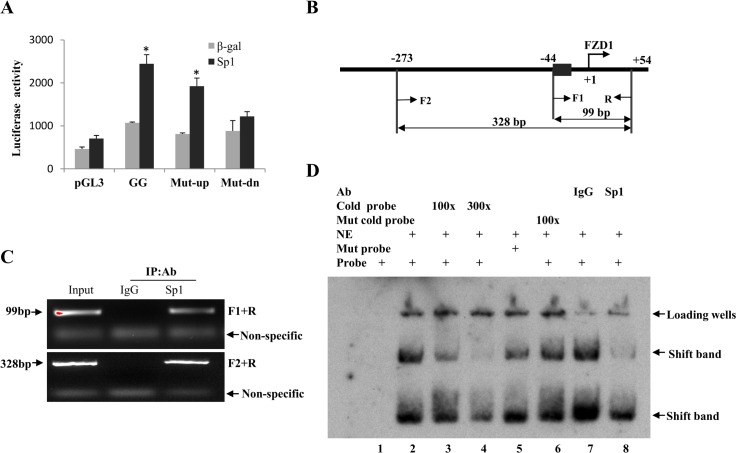
Sp1 binds to the *FZD1* promoter. (A) Sp1 driven increases in the truncated *FZD1* promoter is attenuated by mutation of the putative Sp1 binding site at -44 to -40. Saos2 cells were transfected with truncated wild type promoter, Mut-up or Mut-dn with or without Sp1 expression plasmid. * indicates significant differences in promoter activity between Sp1 and β-gal (*P* < 0.05). (B) ChIP assay of the *FZD1* promoter with antibody specific for Sp1 using Saos2 cells. A schematic representation of the relevant region of the human *FZD1* promoter is shown. F1, F2 and R1 indicate PCR primer pairs used to amplify *FZD1* specific promoter sequences using ChIP DNA. (C) PCR amplification of *FZD1* promoter using ChIP products. The amplified fragments specific for *FZD1* promoter are indicated by arrows. (D) EMSA with probe specific for the putative functional Sp1 binding site located at -44 to -40. Biotin labeled *FZD1* DNA probe was incubated with 2 μg nuclear extracts (NE) from Saos2 cells infected with Ad-Sp1. For the supershift assay, labeled FZD1 DNA probe was incubated with 2 μg NE from Saos2 cells over-expressing Sp1 in the presence of Sp1 antibody (lane 8) or control IgG (lane 7). The locations of the shifted complex bands which were also reduced by Sp1 are indicated as “shifted band”.

To identify the specific Sp1 binding site within the proximal promoter, we performed bioinformatic analysis of the FZD1 promoter and identified two putative Sp1 binding sites at -44 to -40 and -97 to -93 (relative to the translation start site). To characterize these Sp1 sites further, we performed site-directed mutagenesis of these sites using the wild type proximal promoter and co-transfected with Sp1 expression plasmid into Saos2 cells. Mutation of the Sp1 binding site located at the upstream promoter region (-97 to -93) did not affect Sp1 transactivation (Fig 2A Mut-up), whereas mutation of the downstream region (-44 to -40) reduced Sp1 transactivation of FZD1 promoter by 89% (Fig 2A, Mut-dn). To test whether Sp1 binds directly to the FZD1 promoter, we conducted Sp1 specific ChIP assay using Saos2 cells. FZD1 specific products were amplified from the ChIP precipitated DNA using PCR primer pairs spanning either the downstream Sp1 binding site (F1 and R) or both of the putative Sp1 binding sites (F2 and R) (Fig 2B). FZD1 promoter was amplified with both sets of primer pairs demonstrating that Sp1 binds to the -44 to +55 region *in vivo* (Fig 2C). Direct binding of Sp1 to the -44 to -40 region was further confirmed by EMSA analysis with both wild type and mutated oligonucleotides spanning the binding site. Formation of specific binding complexes with the labeled wild type probe were abolished by unlabeled wild type but not mutated probes (Fig 2D). The addition of Sp1 specific antibody dramatically interfered with the formation of Sp1 specific binding complexes, further suggesting that Sp1 binds to this specific site (Fig 2D).

### Sp1 upregulates FZD1 expression

**Fig 3:**
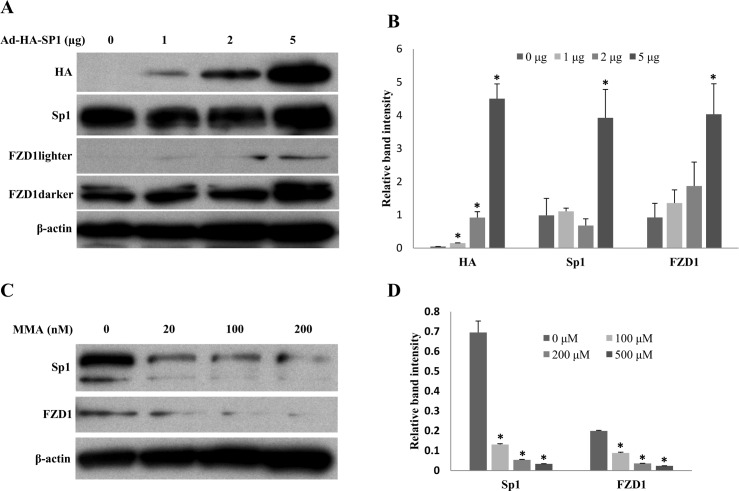
Sp1 regulates FZD1 expression. Over-expression of Sp1 increases FZD1 and MMA treatment decreases both Sp1 and FZD1 expression in Saos2 cells. (A) Dose-dependent up-regulation of FZD1 protein by Sp1. Saos2 cells were infected with the indicated amounts of Ad-HA-Sp1. After 36 hours, cells were harvested for protein and Western blot analysis of both Sp1 and FZD1. (B) Densitometry analysis of the relative levels of protein expression in Fig 3A. (C) Down-regulation of FZD1 expression by Sp1 knockdown in Saos2 cell by MMA. Saos2 cells were treated with MMA at 20, 100, 200 nM for 24 hrs. Protein was harvested for Western blot with Sp1 and FZD1 specific antibodies. (D) Densitometry analysis of the relative levels of protein expression in Fig 3C. * indicates significant differences between the indicated groups (*P* < 0.05).

**Fig 4:**
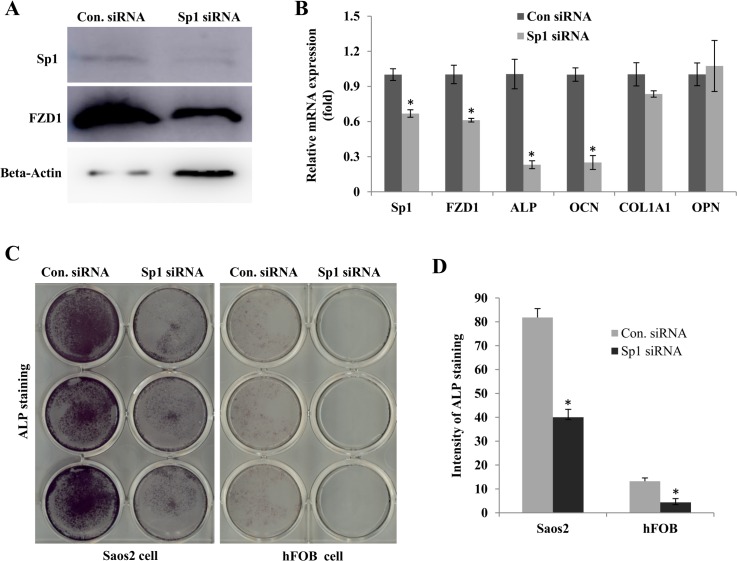
Sp1 knockdown by siRNA in hFOB and Saos2 cells reduces FZD1 expression and osteoblast differentiation. (A) Western blot analysis of Sp1 and FZD1 expression in hFOB cells transfected with 50 nM Sp1 siRNA or scramble siRNA as a control. (B) Real-time quantitative PCR for Sp1, FZD1 and osteoblast differentiation associated genes in hFOB cells transfected with 50 nM Sp1 siRNA or scramble siRNA as a control. (C) ALP activity staining with BCIP/NBT substrate solution (Sigma, USA) in Saos2 and hFOB cells transfected with 50 nM Sp1 siRNA or scramble siRNA. (D) Quantitative results of the ALP staining for Fig 4C by densitometry. All siRNA knockdown experiments were conducted in triplicates and repeated for 2 independent times. * indicates significant difference between the control siRNA and Sp1 specific siRNA treated cells (*P* < 0.05).

To determine whether the Sp1-dependent activation of FZD1 promoter leads to up-regulation of FZD1 expression, we performed Sp1 over-expression or down-regulation experiments in Saos2 cells. Overexpression of Sp1 increased FZD1 expression in a dose dependent manner (Fig 3A). To down-regulate Sp1 protein levels, we treated the cells with MMA, a known antibiotic that down-regulates Sp1 protein expression in different cell types [21–23] and observed dose-dependent decreases in Sp1 protein in Saos2 cells. Consistent with our over-expression experiment, FZD1 protein was also decreased in a MMA dose-depended manner (Fig 3B). Similarly, down-regulation of Sp1 by siRNA in hFOB resulted in lower expression levels of FZD1 and osteoblast differentiation markers, ALP and osteocalcin (OCN), compared to control siRNA (Fig 4A and 4B). Furthermore, ALP activities measured by ALP staining were also reduced in both Saos2 and hFOB cells treated with Sp1 specific siRNA (densitometry intensity 40 versus 82 and 4 versus 13 for Saos2 and hFOB cells, respectively. Fig 4C and 4D). These experiments further support that Sp1 positively regulates the expression of FZD1 and differentiation markers of human osteoblast cells.

### Sp1 alters osteoblast differentiation through regulation of FZD1

**Fig 5:**
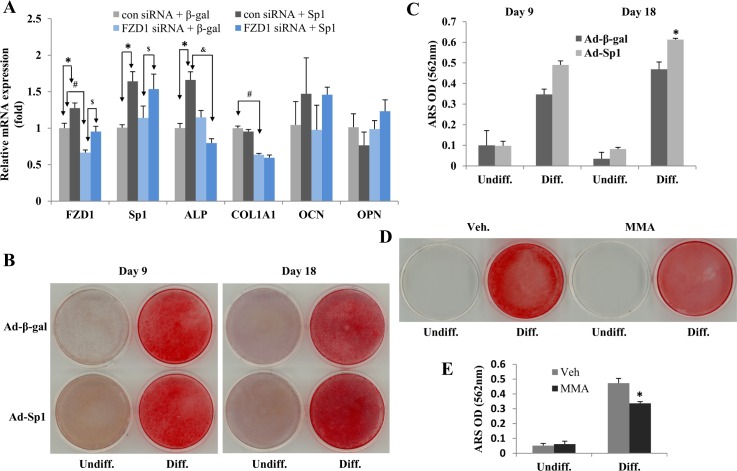
Sp1 regulation of osteoblast differentiation is mediated by FZD1 and Sp1 upregulates Saos2 cell mineralization. (A) FZD1 was knocked down using specific siRNA in Saos2 cells for 24 hr and subsequently transfected with Sp1 or β-gal expression plasmid. Scramble siRNA pre-treated cells were used as a control. RNA was isolated after 48 hr and used for real-time PCR analysis. Gene expression levels in cells treated with specific FZD1 siRNA or scramble siRNA were compared (#). Gene expression levels between cells transfected with Sp1 or β-gal expression plasmid were performed for FZD1 siRNA ($) or scramble siRNA (*) pre-treated cells. The effects of pre-knockdown of FZD1 in Sp1 dependent regulation of gene expression were observed by a direct comparison between FZD1 siRNA and scramble siRNA pre-treated cells which were subsequently transfected with Sp1 expression plasmid (&). P value of < 0.05 was considered to be significant and labeled with each of the above described symbols (#, $, * and &). (B) AR-S staining for mineralization of Saos2 cells over-expressing the Sp1 gene. Saos2 cells infected with Ad-Sp1 or Ad-β-gal were cultured in the presence or absence of osteoblast differentiation medium for 9 days or 18 days, and then the cells were fixed and stained with AR-S. (C) Quantitative results of the AR-S staining for Fig 4A. * indicates significant differences in mineralization between Sp1 and β-gal treated Saos2 cells (*P* < 0.05). (D) Saos2 cells were treated with MMA or reagent control for 24 hours, cultured in differentiation medium for an additional 14 days, and then the cells were fixed and stained with Alizarin Red-S (AR-S). (E) Quantitative results of the AR-S staining for Fig 4C. * indicates significant differences in mineralization between MMA treated and untreated Saos2 cells (*P* < 0.05).

To determine whether the Sp1 effects on osteoblast differentiation were mediated through FZD1, we performed an experiment with a combination of FZD1 knockdown and Sp1 overexpression in Soas2 cells. FZD1 knockdown resulted in significantly lower expression levels of FZD1 and COL1A1 (Fig 5A, #). Overexpression of Sp1 in control siRNA pre-treated cells increased FZD1 and ALP expression levels (Fig 5A,*). However, knockdown FZD1 prior to the overexpression of Sp1 gene abolished the Sp1-mediated effects on ALP (Fig 5A, &). Therefore, Sp1 down-regulation of ALP gene expression appears to be mediated by FZD1.

We have reported that FZD1 is important in osteoblast mineralization *in vitro* using both FZD1 knockdown and overexpression systems [17,18]. Since down-regulation of Sp1 in hFOB cells reduced the expression of both ALP and OCN genes and Sp1 up-regulates FZD1 expression, we tested whether modulating Sp1 expression affects osteoblast mineralization. Mineralization of Saos2 cells was significantly increased at day 18 in cells overexpressing Sp1 compared to cells infected with β-gal control. Interestingly, we did not observe increased mineralization at an early stage of differentiation (day 9, Fig 5B and 5C). Similarly, treatment of the cells with MMA dramatically inhibited the mineralization of Saos2 cells (day 14, Fig 5D and 5E). Furthermore, pre-knock down FZD1 gene reduced the increase of mineralization by Sp1 overexpression, while scramble siRNA pre-treatment did not alter this Sp1 effect in Saos2 cells (data not shown). Thus, our findings suggest that modulation of Sp1 protein expression alters osteoblast differentiation and mineralization *in vitro* through activation of FZD1.

## Discussion

Sp1 is a common transcription factor and plays an important role in cellular growth, cell cycle regulation and apoptosis [1,2,5]. In this study, we discovered that Sp1 is a novel transcriptional activator of FZD1, a co-receptor for Wnt signaling in osteoblasts. We also demonstrated that down-regulation of Sp1 reduced the expression of differentiation markers in both hFOB and Saos2 cells. Furthermore, modulation of Sp1 expression directly affected osteoblast differentiation and mineralization and knockdown of FZD1 prior to Sp1 overexpression abolished the Sp1 effects. Our results suggest that Sp1 plays a novel role in human osteoblast differentiation and mineralization, and that these effects are at least partially mediated by Sp1-dependent transactivation of FZD1.

Sp1 regulates the expression of a number of genes in osteoblasts. For example, podoplanin (PDPN), encoding an integral membrane glycoprotein, is upregulated by Sp1 in MG63, a human osteoblast-like cell line [24]. In Saos2 cells, Sp1 directly binds to the collagen XI alpha 2 (COL11A2) proximal promoter and increases both promoter activity and expression of endogenous COL11A2 [25]. Sp1 also regulates gene expression in rat [26] and mouse osteoblasts [27], bone marrow stromal cells (BMSC) [28], and osteoclasts [27]. In ROS17/2.8, a rat osteoblast-like cell line, Sp1 directly binds to and regulates the PTH/PTHrP receptor gene [26]. In mouse osteoblasts and BMSC, Sp1 regulates the basal transcription of receptor activator of nuclear factor kappa B ligand (RANKL) [28]. The promoter activity and gene expression of integrin β5 are also upregulated by Sp1 in MC3T3-E1 and mouse macrophage cells [27]. Sp1 regulates bone cell differentiation and activity by controlling the levels of transforming growth factor β type I receptor (TGFβ-RI) [29] and regulation of Runx2 expression during osteogenesis [30]. Interestingly, increased Sp1 binding to the type II collagen gene (COL2A1) promoter is required for the stimulation of COL2A1 gene expression by 17β-estradiol in differentiated and dedifferentiated rabbit chondrocytes [31]. Among the Sp protein family, Sp7/Osterix is a widely-studied family member relevant for activation of osteoblast-specific genes and appears to be essential for osteoblast differentiation and bone formation as illustrated in Sp7 knockout mice [8]. In contrast, Sp1 knockout mice die embryonically, making it difficult to explore its function in bone *in vivo [32]*. However, the *in vitro* studies in osteoblasts [24] [25], osteoclasts [27], chondrocytes [31] and BMSCs [28], all suggest a role of Sp1 in bone cell differentiation and bone formation, which is consistent with our results showing that Sp1 increases FZD1 expression and osteoblast mineralization.

Sp1 function is mediated by direct binding to a GC rich DNA sequence [30, 31]. Mithramycin A (MMA) is a GC specific DNA binding antibiotic that inhibits RNA synthesis initiation [33,34]. MMA has been shown to inhibit Sp1 binding to other genes with GC rich promoter motifs [35], and Sp1 expression is also inhibited by MMA in a number of cell types, such as cervical cancer KB cell lines [36], human gastric cancer N87cell lines [37], primary neuronal cells [38] and prostate cancer PC3 and LNCaP cells [39]. In our study, Sp1 protein level was down regulated by MMA in a dose dependent manner in Saos2 cells, which is consistent with results from the above studies. More importantly, MMA treatment decreased both Sp1-dependent activation of FZD1 promoter and upregulation of FZD1 protein expression. Furthermore, MMA treatment also decreased mineralization of differentiated Saos2 cells. Therefore, these results provide additional evidence of Sp1 as a regulator of FZD1 and osteoblast differentiation and mineralization. Since Sp7 binds to similar GC rich sequences in targeted promoters, it will be important to determine whether Sp7 is also a transcription regulator of FZD1 in future studies.

In conclusion, our study demonstrates that Sp1 is a novel and positive transcriptional regulator of FZD1 expression in human osteoblasts. Furthermore, Sp1 regulation of human osteoblast differentiation and mineralization appears to be partially mediated by upregulation of FZD1. Additional studies are needed to dissect the role of Sp1 in the regulation of other FZD family members.
